# Caries of the Teeth

**Published:** 1839-12

**Authors:** Samuel L. Mitchell


### CARIES OF THE TEETH.

On the pathology of this disease, opposite opinions are entertained. Some contend that it is the result of inflammation of the bony structure of the organs; others, that it is that of the action of external corrosive agents; but the arguments and facts that have been brought forward in support of these respectively, it is not our purpose at this time to notice. Our object at present is merely to invite the attention of the profession to the following extract from a letter addressed, by the late distinguished Doctor Mitchell, to Doctor Hope, Edinburgh, in which the latter theory is, in our opinion, established beyond the power of successful refutation. This communication was made in the year 1796, and that it should so long escape the attention of dental writers, is to us a matter of no small surprise. It was by mere accident, that we, while looking over some old volumes of the New-York Medical Repository, a few weeks ago, came across it.

We extract only that part of the letter which has particular reference to this subject. BALT. ED.

*From the New-York Medical Repository, Vol.* 2, 1805.

Application of the doctrine of septic fluids, to explain some of the diseases of human teeth and bones: From a letter of Mr. Mitchell, to Thomas Charles Hope, M. D. &c. Adjunct Professor of Chemistry in the University of Edinburgh: dated, Schenectady, Oct. 10, 1796.

It gives me great satisfaction to learn from your letter of September the 5th, dated Edinburgh, "that you very much agree with me in the views I have adopted, respecting the generation of the varions combinations of oxygen and septon;" that you may feel interested in the progress I may make in the inquiry, and have pleasure in collecting and communicating any thing relative to it that may occur. As you suggested, the pamphlet in which the experimets made to destroy contagion in the British and Russian ships, by means of the vapours emitted from salt-petre, had reached me before the receipt of your letter; and had attempted to show in what manner "the facts might be entirely reconciled with the opinion" which, as you politely observe, so many concurrent circumstances have lead me to adopt. I rejoice that these experiments have been made, and by public authority, upon so large a scale. I hope they will be repeated until complete satisfaction shall be obtained of their true nature and value. From summing up the whole evidence in the most impartial manner I am able, I am induced to believe the greatest part of the benefit derived from them arose from a ventilation, purification, removal of nuisances, and the better medical and dietetic management of the sick; and the rest of the good experienced from the *vital air* extracted from the nitre. The *nitrous gas* appears to have been so far from doing any good, that I am confident the result would have been more clear and decisive without it. I should, therefore, propose, as an amendment of the project, to perform all that was performed on those occasions, excepting the septic gas, and improve the respirability of the air in the apartments, by supplies of *oxygenous gas* alone.

Since the date of my last, I have no reason to retract any part of the doctrine then advanced; but on the contrary, there has been such an influx of new matter, that I have found a necessity of enlarging and extending in all directions. For want of something better, I now take the liberty of presenting you with the application of it, to explain some of the morbid changes which the teeth and bones of animals undergo in the progress of life. It seems to me that we are far enough advanced in knowledge, to attempt something like a scientific explanation of some of the alterations which bones suffer by disease; and this explanation will be the more seasonable, as the writers on surgery have scarcely undertaken any thing of the sort. What I have aimed at in this respect, is to connect the facts related by the late Mr. J. Hunter, of London, with those detailed by Mr. Russel, of Edinburgh, by means of certain experiments and observations of my own.

From the known disposition of oxygen to combine with the septon, and form the septic acid, it appeared to me probable, that it would be formed occasionally in the human mouth, from the remains of food adhering to the teeth, sticking between them, and corrupting them. If this was the case, this acid ought to unite with the calcarious earth of the teeth, and form the septite of lime, which might be washed away by the spittle, or, possibly, in some instances, concrete upon the teeth themselves: and, if formed there, the acid might be expected to corrode the enamel, lay bare the bony part and bring on caries; or incrust the inside, irritate the gums and occasion soreness and bleedings.

In order to determine whether these things were so, I procured from a dentist, a quantity of the substance called "the tartar of the teeth," which I supposed might contain some septite of lime; and subjected it to a number of experiments. Having sometime before received complaints from the merchants of Glasgow, of the faulty quality of the pot-ash supplied from the port of New-York; and having been requested by the Chamber of commerce to visit with him, the stores of the inspectors of those articles in the City of New-York, I had collected samples of pot-ash and pearl-ash of the first qualities, with the view of making experiments upon them.

These salts being in their caustic state, had been placed in separate glasses, to attract water and carbonic acid from the atmosphere; and after standing several months, the ferruginous and earthy parts having subsided, beautiful chrystals of the alkali were formed at the bottom of the liquor. A solution of these crystals was made in water that had been boiled some time, to extract its air, and precipitate some of its earth; and to this solution of pot-ash was added a parcel of the yellowish earthy matter, scraped from the teeth, which had been previously reduced to powder in a mortar.

Instantly on mixing them, bubbles of air were set loose and thickly floated on the surface of the mixture; and by their long continuance without bursting, seemed to indicate a sort of tenacity in the fluid, derived from animal mucilage.

The coarser part of the earthy matter, soon sunk to the bottom; but the finer particles took a longer time to settle down; yet in a few minutes, even before the liquor had become clear, a piece of clean paper dipped in the solution, and dried before the fire, deflagrated on being burned, and emitted numerous flashes and sparkles, after the manner of salt-petre, while no such lucid or radiant appearance was evident on setting fire to paper that had been dried after dipping in a solution of the alkali alone. It is not unworthy to remark, that the smell of the mixture was offensive and nauseous, resembling as much as any thing, the nasty odour of ditch and puddle water. On repeating the experiment several times, and in the presence of several persons, the above mentioned appearances were with trifling variations, similar.

The object of inquiry having been less to make an entire analysis of the lapis dentalis, or "tartar of the teeth," than to ascertain whether it contained any *septic* acid, I shall content myself, for the present, with the persuasion that the question is determined in the affirmative, *by the union of the septic acid and of the tartar*, (which would seem to consist partly of the nitrite of lime, combined with animal mucus, &c.) *with the alkali of the mixture into nitre, which deflagrated on being subjected to the operation of fire, with the paper to which it had attached itself.*

In order to apply this principle it must be understood what the component parts of the teeth are. Scheele and Galen seem, as long ago as 1776, to have succeeded in obtaining *phosphoric acid* from the bones of animals, by employing septic (nitrous) acid, while the phosphoric acid was set free. More recently, Bernard (Journal de Physique, Octobre 1781,) obtained phosphoric acid, not only from fossil bones, from those of the whale and sea horse, but from the tooth of the manati, and the grinder of the elephant. It has also been extracted from ivory.

In short, they who have experimentally attended to the subject, have agreed that animal *bones* are chiefly composed of phosphoric acid and calcarious earth, or are phosphates of lime; and that their *teeth* consist in the main of the like materials. Septic acid, therefore, formed in the mouth decompounds teeth upon the same principle that in the eperiments of the Swedish Chemists, it disorganized bone; that is by detaching the phosphoric acid from the lime, and combining itself with that earthy basis. Hunter, (Natural History of Human Teeth, p. 125,) has an idea that the concretions of the teeth resemble the intestinal balls and bezoars found in the bowels of many animals; and this opinion is probably very just; but perhaps less just is his other idea, that these extraneous matters consist merely of earth and the common secreted mucus. (Diseases of the Teeth, p. 66.) He informs us, that he has seen such earthy depositions cover not only the whole tooth, but part of the gums: in this case, there is always an accumulation of a very putrid matter, and frequently a considerable tenderness and ulceration of the gums, &c.

The destruction of the enamel and bony part of the tooth, the rottenness of alveolar processes, the ulceration, absorption and bleeding of the gums, and the fœtid breath, seem to arise occasionally from the same general cause. To understand the reason of these things, it will be necessary to examine what is the order of chemical elective attraction between phosphoric acid, one of the constituent parts of the teeth, and other bones on the one hand; and between lime, the other ingredient, and the substance with which it has a disposition to combine, on the other.—And in doing this, there will be little or no danger of encroaching upon the vital economy or animated structure of these parts, because the enamel has no marks of being vascular,—and having a circulation of fluids.

Natural History, &c. p. 35.) And there are great doubts whether ever the bony parts possess either blood vessels or absorbents. (Ibid. p. 39.)

Phosphoric acid prefers lime to alkalis, and therefore alkalis united with it are immediately rendered turbid by lime-water, and a saline powder, very difficultly soluble in water, is deposited, consisting of lime saturated with phosphoric acid. Alkalis, therefore, whether fixed or volatile, would seem to be incapable of destroying the solid matter of the teeth, whatever their action may be upon the gums. Lime may be disengaged from its connexion with phosphoric acid by the oxalic, sulphuric, septic (nitric) and tartaric acids. Consequently, acid of sugar, spirits of vitriol, aqua fortis, and cream of tartar may decompose teeth by attracting the lime, and disengaging the phosphoric acid. The septic acid, after barytes, pot-ash, and soda has the next strongest attraction for lime; and after this, for magnesia, ammoniac, and clay.

Septic acid, thus, if formed from the remains of animal and vegetable substances, lurking about or among the teeth, in attaching itself to the lime will detach the acid of phosphorus.

This, added to the matters already emitting their scents, will have a tendency to increase the offensiveness of the breath.

Whatever contributes to the accumulation of the matters from which septic acid is produced, may be expected to injure the teeth. Hence lying-in women, and persons suffering long fits of sickness, are particularly exposed to the causes which destroy them; and this the more rapidly, because in such situations, it often happens that little or no assistance is afforded by art in removing those things, which by their presence and accumulation occasion the mischief. When formed in the mouth, it may mingle likewise with the spittle and vitiate the *gustatory powers of the tongue and palate*, and when swallowed, may impede the healthy *functions of the stomach.* Hence may be explained one species of anorexia, especially that mentioned by Darwin, (2 Zoonomia, class 11, ord. 2, gen. 2, spr. 1,) where want of appetite is sometimes produced by the putrid matter from many decaying teeth being perpetually mixed with the saliva, and thence affecting the organ of taste, and greatly injuring the digestion.

The formation of such a substance in the mouth, enters deeply into the explanation of the symptoms of fevers, particularly the condition of the teeth, gums, tongue and throat; with vitiated taste, thirst, apthæ, color of the tongue, &c.

If an incrustation, containing septic matter is thus formed, and is a calcareous composition, of a kind different from the teeth, it is possible to remove it by chemical agents, which have not the power of decomposing the teeth. For as barytes, pot-ash, and soda, can take the *septic acid* away from the lime, either of these substances may be serviceable in removing the concretion, and at the same time not endangering the teeth, whose phosphoric acid having a greater attraction for lime than for alkalis, is incapable of being displaced by them. Alkaline dentrifices would therefore appear capable of removing the calcareo-septic incrustations from the teeth, but incapable of corroding the teeth themselves. Deboye observes, that tobacco ashes (Le Pratique de Medicine, de La Riviere, &c. lib. vi. ch. 2,) possesses a surprising (tres-merveilleuse,) power to cleanse and whiten the teeth. The active ingredient must be the pot-ash. The practice of some ladies of New-York confirms this.

As the septite of lime is very deliquescent, there is perhaps, only a moderate portion of the septic acid contained in the strong tartar of the teeth. There is another form in which it is peculiarly destructive. Dentists distinguish tartar into three species, the *yellow*, the *black* and the *green*. Of these, the last is observed to be, by a great difference, the most pernicious. It never forms a crust or petrifaction, but always appears like a *green stain*. The enamel of the teeth beneath it is generally corroded and almost always eaten through or destroyed. This is doubtless, owing to the *septic acid* formed under the edge of the gum, or between the teeth, from the remains of food containing septon, which aided by the heat and moisture of the mouth, affords that poisonous and destructive fluid, by uniting with oxygen. I have no hesitation to believe, that a small portion of this acid, formed thus in the mouth, and adhering to a sound tooth, is the cause of that violent and sometimes fatal disease consequent upon transplanting these bony substances into the bleeding jaws of another person. The cases are seldom or never venereal.

But the septic acid, formed in these instances, by corruption on the surface of a tooth, poisons the patient or the dentist, when inserted into the fresh socket, in the same manner that it poisons dissectors, when their wounded fingers receive it from the surface of a putrifying muscle. The safe method of preparing teeth for transplanting, is to wash them repeatedly before extraction, with a weak solution of carbonate of pot-ash, in water to remove the septic acid and other foul matters. This is doubtless, preferable to washing the tooth in alkaline ley after it has been drawn, as thereby its capability to grow fast would be endangered.

The septic acid being thus capable of corroding the teeth and alveolar processes of the jaws, who shall affirm that its operation stops there ? Is it not taken in with our air, food and drink ? Are there not instances of nodes and excresences of bones, that are not wholly unlike the incrustations of the teeth ? And are there not likewise instances of caries of the osseous parts, which have a near similitude to the rottenness of the instrument employed in chewing our food ?

But this opinion is countenanced by better evidence than the poets. For in addition to the diseases of the teeth and their sockets from septic acid produced in the mouth, as already stated, and partly upon the interpretation of the facts of Mr. Hunter, it would be very easy to quote many authorities in the books. Instead, however, of displaying much reading I shall content myself with referring to the authority of an intelligent and skilful surgeon, Mr. Russel, who published, in 1794, a practical essay on that disease of the bones, which is termed necrosis, wherein a bone or part of a bone dies, and a new one is reproduced to supply its place or serve as a substitute. The lower jaw bone is frequently disordered from diseases in the teeth and gums, which, from their situation, naturally determine the complaint to begin at the upper part, and to proceed downwards, (p. 80,)—and necrosis of the lower jaw may be traced to the effects of blows and of tooth ache, especially if a violent attack of inflammation has been excited by the application of any acrid substance to a carious tooth, &c. (p. 98,—and seldom happens to persons under thirty years of age, (p. 93.)

It would seem, therefore, that the reasoning would be fair and safe to consider this caries of the maxillary bone as of a nature quite similar to the decomposition of the teeth and disordered condition of their sockets, which has been shown to be connected with the formation of septic acid in the mouth, and its corroding effects there.

The fœtor of the breath has an analogy, in such cases, with the offensiveness of matter discharged from diseased bones, which in general is discolored and not thick, owing to a separation of the phosphoric acid of the bone from its calcareous base, which is now combined with septic acid, and running away with the other fluids, in the form of a thin and sanious discharge; or occasionally bringing on, when absorbed, that form of quotidian intermitting fever, called the hectic, as in other cases of abscess and ulcer.

Russel considers necrosis, whether happening in the tibea, femus, lower jaw, humerus, fibula, radius, or ulva, as the same kind of disease, (p. 86.) If, then this classification of morbid affections is correct and the application of the principle to explain the phenomena is accurate, it will be proved that the same septic poison which destroys the teeth, corrupts the jaw; and from the same cause which disorganizes the jaw proceeds the decay of the rest of the bones; and this same agent, which works the destruction of the rest of the bones, is the irritating matter that kindles up the hectic fever.

I would not wish to be understood as affirming that all caries of the teeth, jaws, and bones, arise from this sole cause. Far from it. The amount of my reasoning is simply this,—that from the most accurate survey I have been able to take of the subject, there does appear to me to be, in some instances, a decomposition of bone by means of septic acid absorbed from without, or formed, by union of septon with oxygen, within the constitution; and when this acid, mingled with other animal fluids, is carried into the blood vessels, and exerts its noxious powers upon the heart, brain and lungs, it may be the cause of febrile inquietude.

How far this principle may extend, if properly applied I know not. I suspect that syphilitic, cancerous, and scrofulous ulcerations will be found to have a near alliance as to their cause, with caries of teeth, and necrosis of bone now under consideration. But time, with further observation and experiment, is necessary to refute or verify this conjecture. Be the result what it may, I think, as the game is started, and the track is fresh and warm, I would betray less than a sportsman's spirits to be discouraged on account of the doublings and windings of the chace, and give out before the object of pursuit is hunted down, or day light let in upon its dark abode.

But I must quit the subject, and prepare to take my departure from this town, in which, notwithstanding the engagement of my mind upon what is here, you observe I can devote a few minutes to a correspondent in Europe.

To have an idea of the state of things at this place, picture in your mind a settlement, the third in size in the state of New-York, situated in a level spot, beside the river Mohawk, and not far above its greatest cataract; fancy to yourself the chief impediments to navigation removed and the river covered with boats, loaded with families migrating westward to the unsettled lands, or with fruits of agricultural industry coming to market; imagine the goers and comers of gay people, and valetudinarians along this thoroughfare, to the acidulous and medicinal springs of Ball's Town, and the employment of the sons of learning lately established here, in attending to such branches of knowledge, as boys, usually, are taught in colleges : but do not imagine these things and others of a beautiful——. Farewell, and remember I expect from you, as one of the friends and companions of my earlier days, all the help you can afford me in this inquiry.

Yours, with esteem,

Samuel L. Mitchell.

